# TEAD inhibitors re-sensitize drug-resistant NF1 MPNST cells to MEK inhibitors

**DOI:** 10.17912/micropub.biology.002161

**Published:** 2026-07-29

**Authors:** Yang Yang, Jianman Guo, Sharavana Gurunathan, Shane Schechter, Jeffrey Field

**Affiliations:** 1 Systems Pharmacology and Translational Therapeutics, University of Pennsylvania, Philadelphia, PA, US; 2 Harvard University, Cambridge, MA, US

## Abstract

Neurofibromatosis type 1 (NF1) patients develop non-malignant neurofibromas that can progress to Malignant Peripheral Nerve Sheath Tumors (MPNSTs). NF1 neurofibromas are treated with MEK inhibitors, such as mirdametinib and selumetinib, because they are driven by activation of the Ras/Raf/MEK/ERK signaling pathway. We developed two MEK-resistant MPNST cell lines by passaging cells in selumetinib for approximately three months. The cells were resistant to 15 other MEK inhibitors in a high-throughput screen but were re-sensitized by co-treatment with a TEAD inhibitor. These results suggest that TEAD inhibitors synergize with MEK inhibitors to overcome resistance and enhance therapeutic efficacy in MPNSTs.

**Figure 1. MEK inhibitor-resistant MPNSTs are re-sensitized by TEAD inhibitors:** (A) IC _50_ heatmap of 130 drugs profiled against parental ST88-14, ST88-14 MR II, and ST88-14 MR II co-treated with 5 µM of the TEAD inhibitor VT103. Dose-response curves for trametinib (B), selumetinib (C), and VT103 (D) for parental ST88-14 and ST88-14 MR I and II cells. (E) Synergy ZIP score averages of the parental ST88-14 and ST88-14 MR I and II cell lines subjected to two-drug combination treatments. Drug-drug interactions were quantified with SynergyFinder Plus using the Zero Interaction Potency (ZIP) model. For each drug pair, cells were treated with each agent alone and in combination across a matrix at six concentrations from 5 nM to 5 μM for trametinib and 10 nM to 10 μM for VT103. Cell viability was measured after treatment and dose-response curves were generated for both single agents and combinations. The ZIP model evaluates synergy by comparing the observed combination response to an expected response assuming the two drugs do not affect each other’s potency. Deviations from this expected additivity are integrated across the full dose-response surface to generate a ZIP synergy score. ZIP scores greater than 10 were interpreted as strong synergy, scores between −10 and 10 as additive or independent effects, and scores less than −10 as antagonism. Average ZIP scores across the dose matrix were used to summarize drug interactions. For the VT103 + Trametinib combination, we performed three one-way ANOVA tests between each cell line. Comparing the parental ST88-14 ( *n* = 7) and ST88-14 MR II ( *n* = 3) cell lines, a one-way ANOVA yielded no statistically significant difference between the groups ( *p* = 0.1888). For ST88-14 and ST88-14 MR I ( *n* = 2), a one-way ANOVA revealed no statistically significant difference between the groups ( *p* = 0.6199). Regarding ST88-14 MR I and ST88-14 MR II, a one-way ANOVA revealed a statistically significant difference ( *p* = 0.0270).

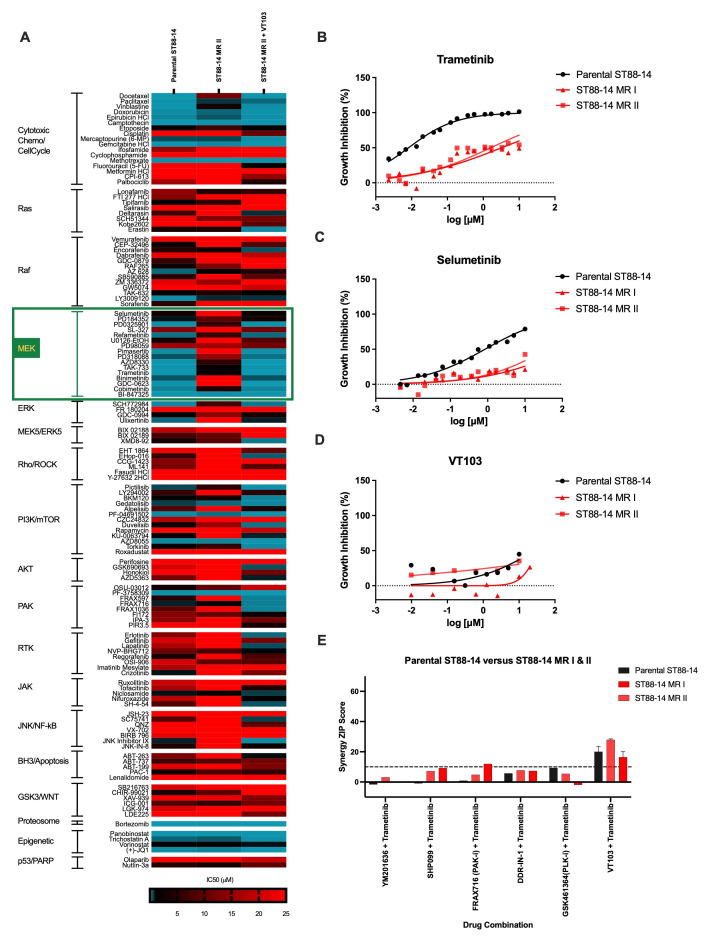



## Description

NF1 is a tumor syndrome characterized by non-malignant tumors of Schwann cells called neurofibromas. In 8 to 13% of patients, neurofibromas progress to life-threatening MPNSTs. The NF1 gene encodes neurofibromin, a negative regulator of Ras, so NF1 loss activates Ras and its downstream signals through Raf/MEK/ERK and other signals such as PI3K/mTOR (Anastasaki et al., 2022). The MEK inhibitors selumetinib and mirdametinib (PD 0325901) are FDA-approved to treat neurofibromas, and trametinib was effective in a small-scale clinical trial (Gross et al., 2020; Kiaei et al., 2022; Moertel et al., 2025). However, MEK inhibitors are not effective as single agents in MPNST models. In addition, patients showed low response rates in a recent clinical trial of a combination of a MEK inhibitor with an mTOR inhibitor (de Blank et al., 2022; Kim et al., 2026). Therefore, there is a clinical need for MPNST therapies, especially in combination with MEK inhibitors.

Because MEK inhibitors are used to treat neurofibromas, many MPNSTs are likely to present in the clinic with preexisting MEK inhibitor resistance. Amplification of mechanosignaling pathways, including Pak/Rac and Hippo/YAP-TAZ/TEAD, can drive MEK inhibitor resistance in melanomas and pancreatic cancer (Edwards et al., 2023; Lin et al., 2015; Long et al., 2026; Lu et al., 2017). Although YAP-TAZ signaling has been implicated in MPNSTs, its role in MEK inhibitor resistance has not been explored extensively (Wu et al., 2018; Yang et al., 2026). A new class of compounds inhibits Hippo signaling by binding to the auto-palmitoylation pocket in TEAD, the YAP/TAZ partner (Tang et al., 2021). These compounds have shown some clinical efficacy in NF2-dependent mesotheliomas (Yap et al., 2025), where TEAD is likely to be a major tumor driver. TEAD inhibitors also showed some efficacy in preclinical models of another form of neurofibromatosis, *NF2* -SWN, either alone or together with PAK inhibitors (Benton et al., 2024; Laraba et al., 2023; White et al., 2019; Yang et al., 2026). We hypothesized that, even if MEK inhibitor-resistant, NF1-MPNST will respond to dual inhibition with combinations of a TEAD inhibitor plus other inhibitors of Ras signals, such as MEK and mTOR inhibitors.

To test this hypothesis, we first developed MEK inhibitor-resistant MPNST cells. Three cell lines that have been extensively characterized by genomic analysis, ST88-14, sNF96.2, and sNF02.2, were authenticated by Short Tandem Repeat (STR) profiling and then subjected to selumetinib passaging. The cells were serially passaged in the presence of selumetinib for ~3 months until they developed resistance. After approximately 6 passages in drug-free media, cells were tested with selumetinib and trametinib to determine sensitivity. ST88-14 cells rescreened resistant, but sNF02.2 and sNF96.2 did not yield stable resistant isolates. Thus, resistance modeling was discontinued with sNF02.2 and sNF96.2 cell isolates. We performed selection twice, yielding two selumetinib-resistant cell lines, ST88-14 MR I cells and ST88-14 MR II, and then passaged the cells 8 more times at high doses to confirm resistance. The ST88-14 MR I cell line was authenticated again via STR profiling, which found 100% similarity to its parental ST88-14 reference, indicating no gross chromosomal changes from extended growth in selumetinib.

We next performed a high-throughput screen against a library of ~130 drugs, at eight concentrations of each drug ( Figure 1, Panel A) (Guo et al., 2017). The drug library was developed specifically for NF and contains a comprehensive set of Ras pathway drugs with 16 MEK inhibitors including selumetinib, mirdametinib, and trametinib. The IC _50_ values for parental and resistant ST88-14 MR II cells are shown in the first two columns of Panel A. Many drug sensitivities were unaffected, but the cells were markedly resistant to 15 of the 16 MEK inhibitors tested, with substantial increases in IC _50_ values. The otherwise MR cell lines continued to be sensitive only to BI-847325, but this drug also targets Aurora kinase, a known MPNST target (Patel et al., 2012). Resistance also developed to other classes of Ras inhibitors, including ERK, mTOR, and RTK inhibitors.

Resistance stability was confirmed by retesting in triplicate for sensitivity to trametinib (Panel B) and selumetinib (Panel C). In the MEK inhibitor-sensitive parental ST88-14 cell line, the IC _50_ values of trametinib and selumetinib were 0.008 μM and 0.88 μM, respectively. Both values were higher in the resistant cell lines: 3.29 μM (ST88-14 MR I) and 1.95 μM (ST88-14 MR II) for trametinib, and 25 μM (ST88-14 MR I) and 23 μM (ST88-14 MR II) for selumetinib. Dividing the IC _50_ of the resistant cell line by the IC _50_ of the sensitive cell line computes the resistance index (RI). The RIs for trametinib were 406 and 239 in ST88-14 MR I and ST88-14 MR II, respectively, compared to 28.4 and 26.1 for selumetinib. The dose-response curve for all three cell lines when treated with VT103 alone is shown in Panel D.

Given evidence in melanoma and pancreatic cancer that YAP-TAZ/TEAD can drive resistance to Ras/Raf/MEK inhibitors, and that YAP-TAZ inhibition synergized with Raf inhibition in MPNSTs, we tested a role for TEAD. We co-treated resistant cells with the TEAD inhibitor VT103 plus each of the ~130 drugs in our library ( Figure 1, Panel A, 3 ^rd^ column). VT103 re-sensitized MEK inhibitor-resistant cells to all previously ineffective drugs, including all 15 MEK inhibitors, PAK inhibitors, and mTOR inhibitors. To quantify re-sensitization, we performed synergy analysis of MEK and TEAD inhibitor pairs and several other pairs of NF1-relevant drugs. The synergy analysis was performed by dosing two drugs alone and in combination in microtiter plates. We tested five to seven doses of each drug at concentrations centering on the IC _50_ values determined from Panel A. Synergy was analyzed using SynergyFinder to quantify the synergy between drug pairs (Zheng et al., 2022). SynergyFinder’s Zero Interaction Potency (ZIP) model compares changes in potency across dose-response curves for single drugs and drug combinations to determine drug interaction relationships. Synergy ZIP scores greater than 10 indicate that two drugs, when used in combination, are highly synergistic. We found that combinations of TEAD and MEK inhibitors were highly synergistic in the parental cells, and comparably synergistic in the resistant cells ( Figure 1, Panel E). High levels of synergy were also seen with other TEAD inhibitors and when data were analyzed using the Chou-Talalay method with CompuSyn software (Chou & Talalay, 1984).

MEK inhibitors are a major clinical advance as the first FDA-approved drugs to treat neurofibromas. However, they perform poorly in MPNSTs as single agents and in combination with mTOR inhibitors. Additionally, results with other malignant tumors, such as melanoma, indicate that MEK inhibitor resistance may develop within months. YAP-TAZ signal amplification has been implicated in MEK-sensitive tumors, and, in some cases, activation can promote MEK inhibitor resistance. Prior studies have mostly used genetic ablation or expression of dominant-negative YAP-TAZ mutants because YAP and TAZ have been difficult to target with drugs. Fewer studies have addressed whether resistance can be prevented by TEAD inhibition. Our studies suggest prioritizing testing TEAD inhibitors in combination with MEK inhibitors for further preclinical testing.

**Limitations of this study:** Cell-based screening studies can effectively identify promising drug combinations as starting points for subsequent animal and, potentially, clinical evaluation. However, only 5–10% of cell and animal studies translate into clinical success. For instance, the combination of MEK and mTOR inhibitors was cytostatic in MPNST animal models and induced tumor shrinkage yet provided minimal benefit in patients (Kahen et al., 2018; Kim et al., 2026). A limitation of our dataset is its reliance on a single parental cell line background: ST88-14. Stable resistant isolates could not be established from sNF02.2 or sNF96.2 cells; thus, all resistance data were generated exclusively in the ST88-14 cell line. Despite these caveats, the combination of the MEK inhibitor trametinib demonstrated comparable synergy with TEAD inhibitors in the sNF02.2 and sNF96.2 cell lines, and another study found synergy with a different class of YAP-TAZ inhibitors plus MEK inhibitors in other MPNST models (McGee et al., 2025). These findings support further preclinical testing of the VT103 TEAD inhibitors in combination with MEK inhibitors as a potential therapeutic strategy for MPNSTs.
